# Supplementary material for: A comprehensive and standardized pipeline for automated profiling of higher cognition in mice
Source: Cell Rep Methods. 2025 Mar 17;5(3):101011. doi: 10.1016/j.crmeth.2025.101011 (PMC12049718; doi:10.1016/j.crmeth.2025.101011)
Supplement: Document S1. Figure S1 [file mmc1.pdf]

Cell Reports Methods, Volume *5*

Supplemental information

A comprehensive and standardized pipeline for automated profiling of higher cognition in mice

Vinicius Daguano Gastaldi, Martin Hindermann, Justus B.H. Wilke, Anja Ronnenberg, Sahab Arinrad, Sabine Kraus, Anne-Fleur Wildenburg, Antonios Ntolkeras, Micah J. Provost, Liu Ye, Yasmina Curto, Jonathan-Alexis Cortés-Silva, Umer Javed Butt, Klaus-Armin Nave, Kamilla Woznica Miskowiak, and Hannelore Ehrenreich

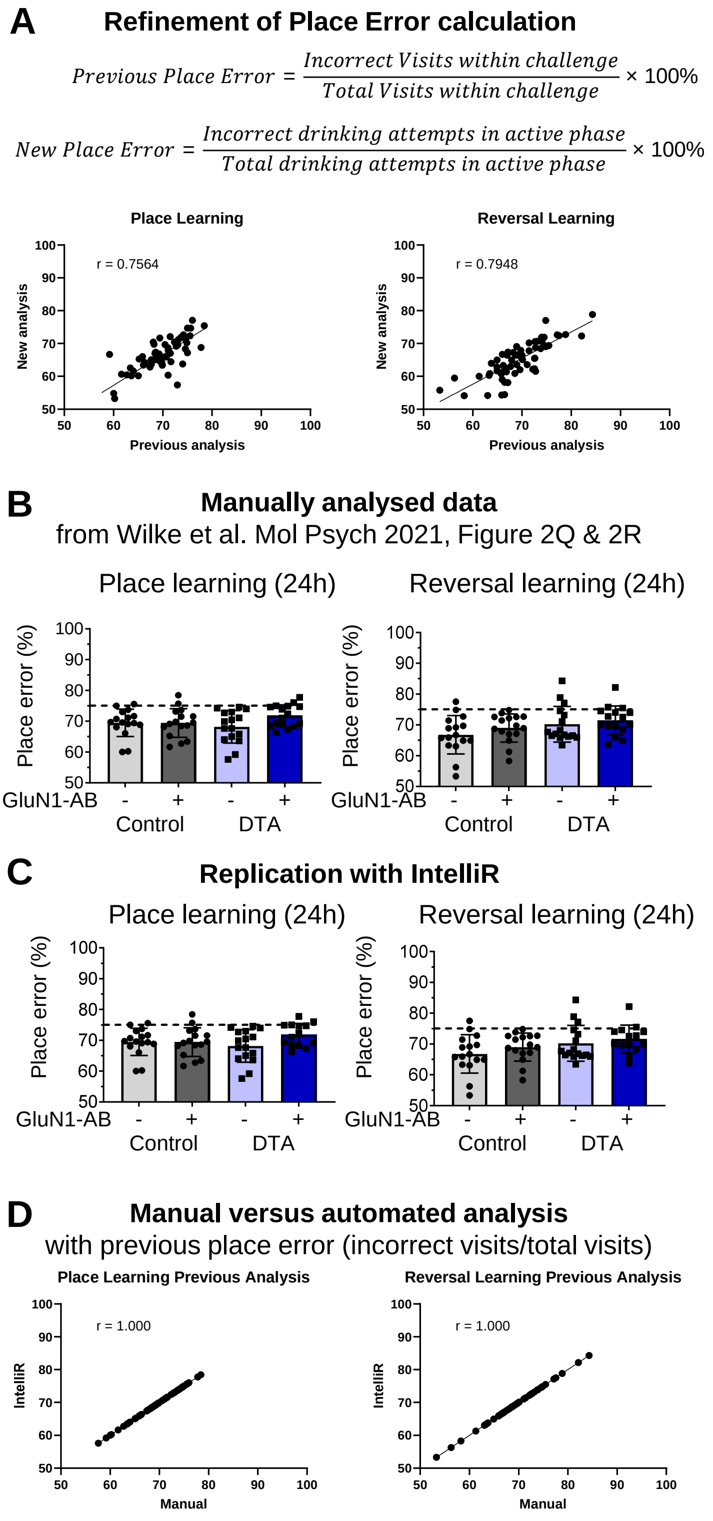

Supplementary Figure 1: Replication of manually analyzed IntelliCage data with IntelliR. (A) Comparison of the previous place error calculation used by Wilke et al. 2021 [S1], that was based on corner visits independent of nose pokes and the new place error calculated by IntelliR, that distinguished between exploratory visits and drinking attempts (visits with nose poke). While Pearson correlations of the previous and new place error calculation show substantial correlation between the old and new analysis (r=0.75-0.79), they also reveal differences, likely due to the exclusion of exploratory visits in the new Place Error. Hence, to test if automated data analysis yields the same results as manual analysis, we implemented the previous place error calculation into IntelliR and re-analyzed the data published by Wilke et al. 2021 [S1] **(B-C)**. **(D)** Pearson correlation analysis of manually and automatically analyzed IntelliCage data demonstrate perfect replication of manually analyzed data with IntelliR (r=1.0). Related to Figures 2, 4, and 5.

Reference:

[S1] Wilke, J.B.H., Hindermann, M., Berghoff, S.A., Zihsler, S., Arinrad, S., Ronnenberg, A., Barnkothe, N., Steixner-Kumar, A.A., Roglin, S., Stocker, W., et al. (2021). Autoantibodies against NMDA receptor 1 modify rather than cause encephalitis. Mol Psychiatry 26, 7746-7759. 10.1038/s41380-021-01238-3.
