# Supplementary material for: A comprehensive and standardized pipeline for automated profiling of higher cognition in mice
Source: Cell Rep Methods. 2025 Mar 17;5(3):101011. doi: 10.1016/j.crmeth.2025.101011 (PMC12049718; doi:10.1016/j.crmeth.2025.101011)
Supplement: Document S2. Article plus supplemental information [file mmc2.pdf]

Cell Reports
Methods

Article

A comprehensive and standardized pipeline for automated profiling of higher cognition in mice

Graphical abstract

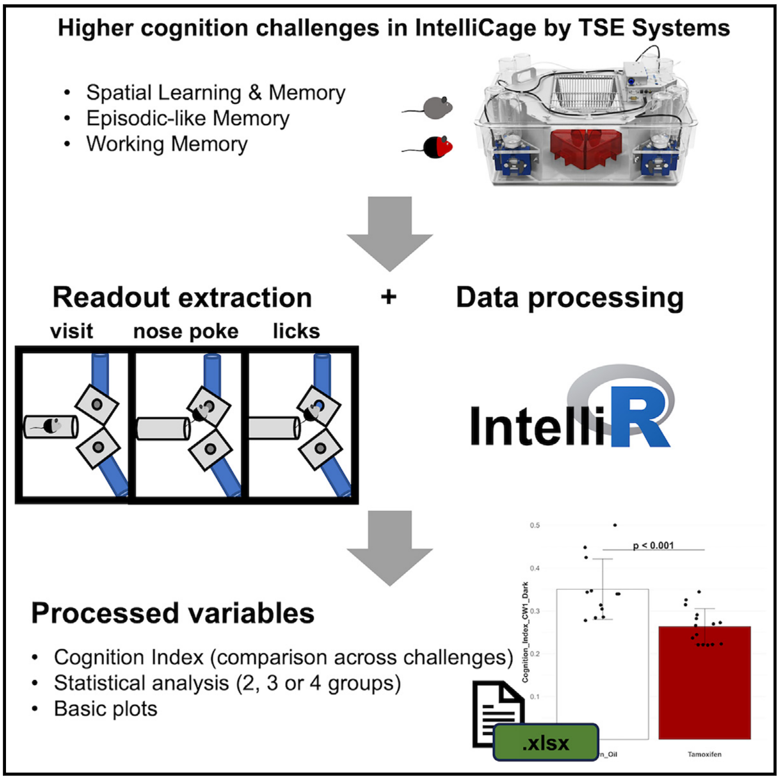

Authors

Vinicius Daguano Gastaldi,
Martin Hindermann, Justus B.H. Wilke, ...,
Klaus-Armin Nave,
Kamilla Woznica Miskowiak,
Hannelore Ehrenreich

Correspondence

hannelore.ehrenreich@web.de

In brief

Gastaldi et al. introduce an open-source analysis pipeline for IntelliCage data that can process and analyze different challenges. Additionally, they provide three challenges that evaluate important cognitive domains in neuropsychiatric disorders. These are learnable by healthy mice and uncover a decline in working memory in mice with ablation of pyramidal neurons.

Highlights

- IntelliR provides a free and standardized pipeline for analyzing IntelliCage data
- IntelliR challenges evaluate spatial, episodic-like, and working memory with reversals
- Cognition index permits comparison of performance across cognitive domains
- Mice with ablation of pyramidal neurons decline mainly in working memory

Daguano Gastaldi et al., 2025, Cell Reports Methods 5, 101011
March 24, 2025 © 2025 The Author(s). Published by Elsevier Inc.
https://doi.org/10.1016/j.crmeth.2025.101011

CellPress

Cell Reports Methods

CellPress
OPEN ACCESS

Article

A comprehensive and standardized pipeline for automated profiling of higher cognition in mice

Vinicius Daguano Gastaldi,[1,2,7] Martin Hindermann,[1,2,7] Justus B.H. Wilke,[1,7] Anja Ronnenberg,[1] Sahab Arinrad,[1] Sabine Kraus,[1] Anne-Fleur Wildenburg,[1,3] Antonios Ntolkeras,[1,2] Micah J. Provost,[1,2] Liu Ye,[1,4] Yasmina Curto,[1] Jonathan-Alexis Cortés-Silva,[1] Umer Javed Butt,[1] Klaus-Armin Nave,[3] Kamilla Woznica Miskowiak,[5] and Hannelore Ehrenreich[1,2,6,8,*]

[1]Clinical Neuroscience, Max Planck Institute for Multidisciplinary Sciences, City Campus, 37075 Göttingen, Germany
[2]Georg-August-University, Göttingen, Germany
[3]Department of Neurogenetics, Max Planck Institute for Multidisciplinary Sciences, City Campus, 37075 Göttingen, Germany
[4]Department of Anesthesiology, Guangxi Medical University Cancer Hospital, Nanning 530022, China
[5]Neurocognition and Emotion in Affective Disorders (NEAD) Centre, Copenhagen Affective Disorder Research Centre, Psychiatric Centre Copenhagen, Frederiksberg Hospital and Department of Psychology, University of Copenhagen, 2000 Frederiksberg, Denmark
[6]Experimental Medicine, Department of Psychiatry and Psychotherapy, Central Institute of Mental Health, Medical Faculty Mannheim, Heidelberg University, J 5, 68159 Mannheim, Germany
[7]These authors contributed equally
[8]Lead contact
*Correspondence: hannelore.ehrenreich@web.de
https://doi.org/10.1016/j.crmeth.2025.101011

MOTIVATION Mouse models and the analysis of their behavior are an essential part of research in neuropsychiatric diseases. IntelliCages are a powerful system for automated behavioral testing that have transformed the landscape of behavioral research in rodents. Although numerous research articles have been published using this system, there is significant variability when it comes to the analysis of its output. Here, we present IntelliR, a free and open-source standardized pipeline that can be used to consistently analyze output data from IntelliCages and can be adapted to any design. It differentiates itself as it is mostly automated, requiring minimal input from the researcher to run. We also introduce a set of three challenge designs that cover spatial, episodic-like, and working memory, crucial cognitive domains in neuropsychiatric research.

SUMMARY

In rodent behavior research, observer-independent methods, such as the IntelliCage, enhance data collection in a social, and thus stress-reduced, environment. The IntelliCage system allows experimenters to create cognitive challenges for mice motivated by rewards. Given the extensive and diverse data from IntelliCage, there is a high demand for automated analysis. Here, we introduce IntelliR, a free and standardized pipeline for analyzing IntelliCage data, including a cognition index for performance comparison across challenges. IntelliR supports the automatic analysis of three challenges that cover spatial, episodic-like, and working memory with their reversal tests and can also be adapted for other designs. Results from three cohorts of adult female C57B6 mice showed improved task proficiency over time. To validate cognitive impairment detection, we used adult female NexCreERT2xRosa26-eGFP-DTA mice after neuron ablation in cortex and hippocampus, in which we observed reduced learning capabilities. IntelliR integrates easily into research, improving time management and reproducibility.

INTRODUCTION

The rapid development of elaborate mouse models for neuropsychiatric diseases and human brain pathology created a pressing need for comprehensive and well-standardized high-throughput behavioral phenotyping protocols.[1,2] In response, a variety of standardized scoring systems and behavioral testing protocols have been developed, validated, and proven extremely useful. These methods typically rely on either fully automated equipment, semi-automated tracking software, or

This is an open access article under the CC BY license (http://creativecommons.org/licenses/by/4.0/).

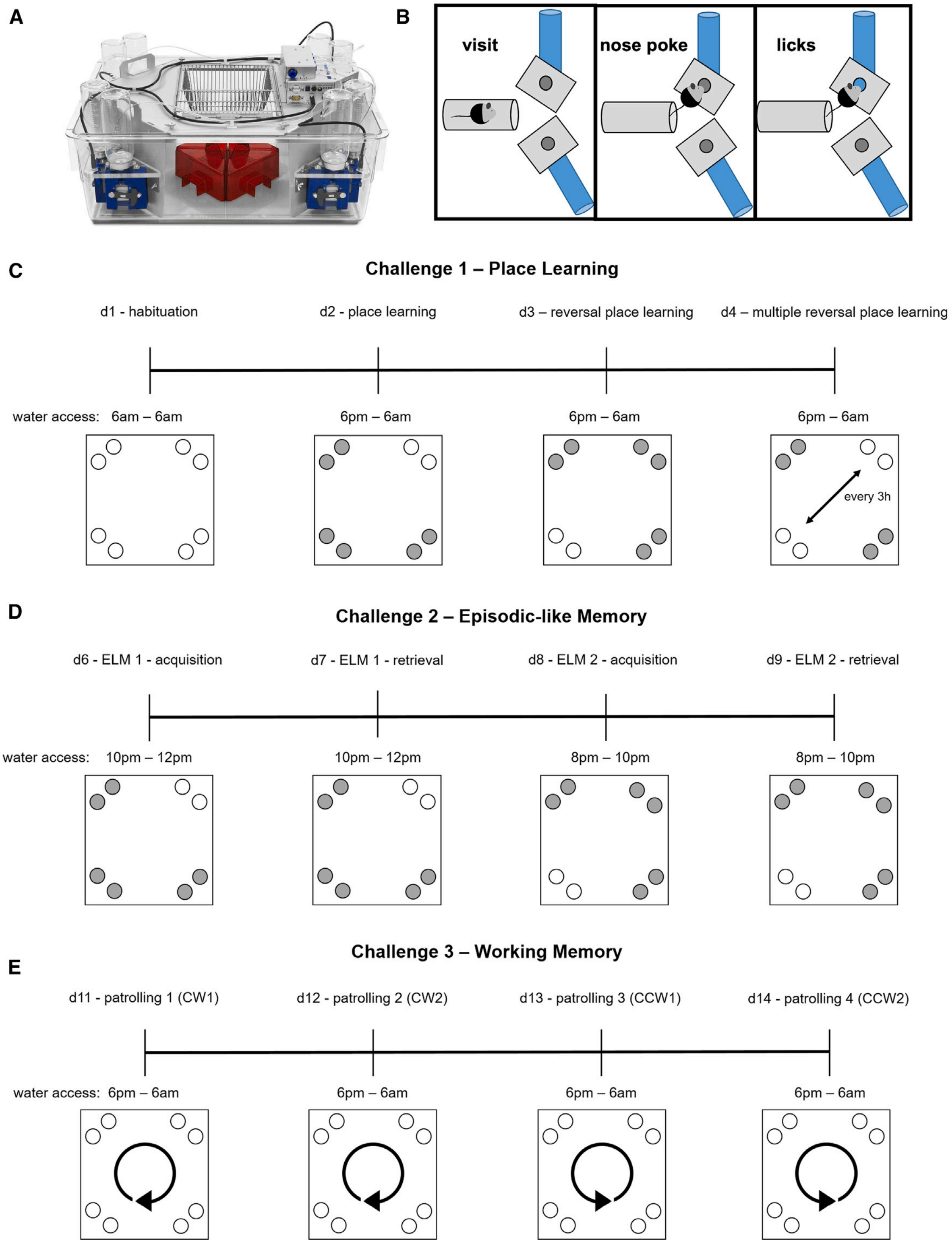

Figure 1. Descriptive overview of the IntelliCage setup and programmed challenges

(A) IntelliCage system for cognitive challenges of up to 16 mice. Conditioning corners allow access to water bottles and are equipped with sensors to detect presence and track behavior of mice within the corners.

(B) Graphical description of the three basic behavior readouts provided by the system: presence of mice within the tube leading to the water bottles (visit), mice touching the door blocking the access to a water bottle with their noses (nose poke), and, after this, the door opens and the mice have access to water bottles and are able to drink (licks).

(C–E) Graphical outline of the programmed challenges representative for one group.

(legend continued on next page)

experimenter-dependent testing and scoring of predefined readouts.[2–4] Yet, despite the attempt to standardize these tests, considerable variability and potential stressors may be introduced by environmental factors, singling of mice for testing, handling, and diverse housing conditions.[1,2] To minimize these problems, the IntelliCage system was developed to enable automated phenotyping of mice in an observer-independent social setting, using longitudinal monitoring and cognitive profiling of individual mice in their home cage amid peers[1,2,5] and has shown great reproducibility across different laboratories.[6,7]

An IntelliCage provides space for up to 16 mice and is equipped with four computer-controlled corners that function as operant chambers with just enough space for a single mouse. Mice are individually tracked by small subcutaneous radiofrequency identification transponders that are registered by antennas within the corner entries. Each corner is equipped with programmable light-emitting diodes, and two drinking bottles with built-in "lickometers" that register contacts with the drinking nipple. Access to the drinking nipples is provided through holes with nose poke sensors and is controlled by motorized doors.[2,5] The corners can be programmed to restrain or enable access to the bottles based on conditions such as time, location of the corner, presence of an operant response (i.e., nose poke), a sequence of events, and more.[1,2,8–10] Dependent on the testing protocol, learning can be reinforced either positively via rewards, such as access to tap water, sucrose, drug solutions, or negatively via aversive stimuli, such as airpuffs, bitter, sour or allergen solutions, or by light-emitting diodes that provide diverse visual stimuli.[2] This versatility facilitated the development of a multitude of IntelliCage-based challenges assessing diverse behavioral domains, including general and circadian activity, exploration, stereotypy, emotionality, social behavior, consumption behavior (i.e., preference/avoidance of drugs or allergens), and, finally, learning and memory.[2] However, despite the increasing number of available IntelliCage-based challenges, data analyses and statistical methods vary considerably across studies[2,5,7,11–18] and often rely on manual data extraction from the graphical user interface of the IntelliCage Analyzer software, which is time consuming and prone to errors.[18]

To improve reproducibility and to overcome the challenges associated with the analysis of large and heterogeneous datasets, we developed the free and open-source IntelliR data analysis platform. IntelliR provides standardized and automated data extraction, data summary, plotting, and statistical analyses for higher cognitive challenges, including the domain place learning, (multiple) reversal learning, extinction, episodic-like and working memory, as well as executive functioning. The platform can be run upon individual challenges and can be modified with minimum R-programming knowledge for the analysis of additional modules. We also introduce conditional parameters that are inaccessible in the default IntelliCage Analyzer software to better assess cognition, exploration, and repetitive behaviors. To validate the IntelliR platform and associated IntelliCage protocols, we confirmed that cognitive challenges are indeed learned by healthy mice, using three independent cohorts. Finally, we employed transgenic mice after sterile induction of pyramidal neuronal death in the cornu ammonis[19–21] and corroborated the ability to assess hippocampus-dependent cognitive function.

RESULTS

The IntelliR pipeline

To standardize and speed up the analysis of heterogeneous IntelliCage datasets, we developed the IntelliR pipeline along with three comprehensive cognitive challenges that assess spatial learning and memory, episodic-like memory, and working memory in a patrolling task (Figure 1). Furthermore, all challenges include reversal tasks to measure cognitive flexibility in assessed domains. IntelliR has an easy-to-use interface that extracts relevant information from IntelliCage experiments within a few clicks and minutes. The code is provided as an R-script, and its basic structure can be easily modified to account for different challenges and experimental settings.

When provided with raw data from the IntelliCage and information about the experiment, i.e., the number and names of groups and an unblinding file, IntelliR extracts "classical" IntelliCage readouts, such as the number of visits, licks, and nose pokes per animal. Additionally, we designed IntelliR to display readouts such as the number of drinking attempts, as well as the errors for place, time, challenge, and direction, which are detailed in the STAR Methods section. For a detailed introduction and demonstration of the usefulness of the classical IntelliCage readouts, we kindly refer to two excellent expert reviews.[2,22] After the data are extracted, the readouts are evaluated at four different time periods. These are the dark phase, the light phase, total 24 h, and an "active" phase, which is the challenge-dependent time window in which mice are required to learn a specific cognitive task and perform accordingly. For each parameter in each time window, IntelliR computes relevant statistical parameters, including the number of animals per group, group means and standard deviation, as well as effect sizes and p values. Afterward, IntelliR summarizes the information of group performances

(C) On day 1 (d1), mice have free access for 24 h to all water bottles in the corners (blue circles) to habituate to the new environment and task to get water. On d2, water access is only available in the dark phase (active phase) and one specific corner per group (place learning). On d3, water access is only in the dark phase and the corner diagonally opposite of the previously correct corner (reversal learning). On d4, during dark phase, correct corner for water access switches every 3 h between the two previously correct corners (multiple reversal learning).

(D) After one day with free access to water for 24 h (extinction, d5), challenge 2 starts with access to water from 10p.m. to 12p.m. in the first of the previously learned corners (episodic like memory–acquisition). On d7, the settings from d6 are repeated (episodic like memory–retrieval). On d8, water access shifts to 8p.m.–10p.m. and to the corner diagonally opposite of the formerly correct corner (episodic like memory reversal–acquisition). On d9, the settings from d8 are repeated (episodic like memory reversal – retrieval).

(E) After another day with free access to water for 24 h (extinction, d10), challenge 3 tests for working memory via the patrolling paradigm. On d11 and d12, water access is granted during active phase and correct corners switch clockwise after each correct drinking attempt (nose poke). The first correct corner is defined by the first drinking attempt of the mice. On d13 and d14, correct corners switch counterclockwise after each correct drinking attempt. First correct corner is defined by the last correct drinking attempt during clockwise patrolling.

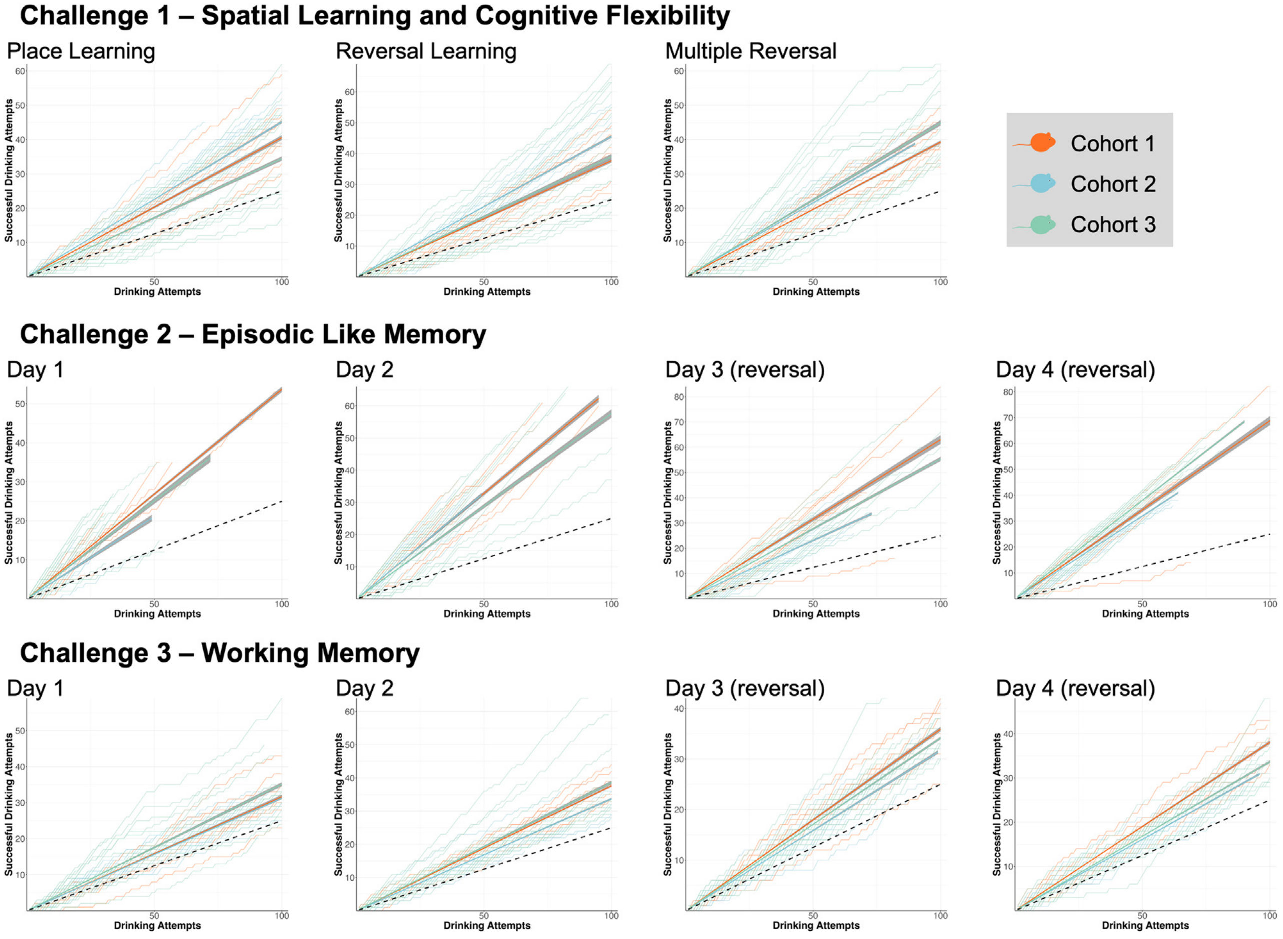

Figure 2. Learning performance of three independent cohorts of healthy female mice in individual cognitive tasks

Cumulative successes at each drinking attempt were plotted for individual mice (faint lines), and group performances calculated by linear regression (bold lines; 95% CI depicted as a shade) were compared to the average by-chance performance (dashed line) through estimated marginal means contrasts. All three independent cohorts of mice successfully learned all cognitive tasks as indicated by significant above-chance performance (all $p < 0.0001$). Only a small number of individual mice failed to learn some of the tests as indicated by close to or below chance performance. Global comparisons were calculated using analysis of covariance (ANCOVA), with specific group comparisons calculated through estimated marginal means contrasts.

and comparisons as well as the performance of individual mice in all readouts within all time periods into csv (or Excel files) and creates bar graphs for quick data inspection. To ensure correct data extraction and processing, we performed a Pearson correlation analysis on place errors computed by IntelliR with place errors calculated by manual data extraction and analysis using a previously published IntelliCage dataset.[21] The resulting Pearson correlation coefficients of 1.0 demonstrated perfect replication of manually analyzed data by IntelliR (Figure S1). In the following, we provide various examples of how IntelliR data can be utilized and validate the three cognitive challenges accompanying the IntelliR data analysis pipeline.

Mice are able to learn all cognitive challenges provided by IntelliR

To confirm that mice perform as hypothesized and properly learn the cognitive tasks, we calculated learning curves of each task for three independent cohorts of healthy female mice tested in the IntelliCages (Figure 2). Comparing the learning curves with the average by-chance performance using estimated marginal means contrasts revealed that all three independent cohorts successfully learned all 11 cognitive challenges as indicated by significant above-chance performance (all $p < 0.0001$). Only a small number of individual mice failed to learn single tasks as indicated by similar to or worse than by-chance performance (Figure 2).

Assessing hippocampal dysfunction using IntelliR

To test whether the cognitive challenges are sensitive enough to detect hippocampal dysfunction, we utilized transgenic diphtheria toxin A (DTA) mice in which pyramidal cell death can be acutely induced via tamoxifen.[19–21] Pronounced neuronal cell death and microgliosis in the cornu ammonis region at the time of IntelliCage testing was confirmed using male DTA littermates, which were treated in parallel to the behavior cohort (Figures 3A and 3B). Within the behavior cohort, prominent atrophy and microgliosis in the cornu ammonis region of tamoxifen-induced

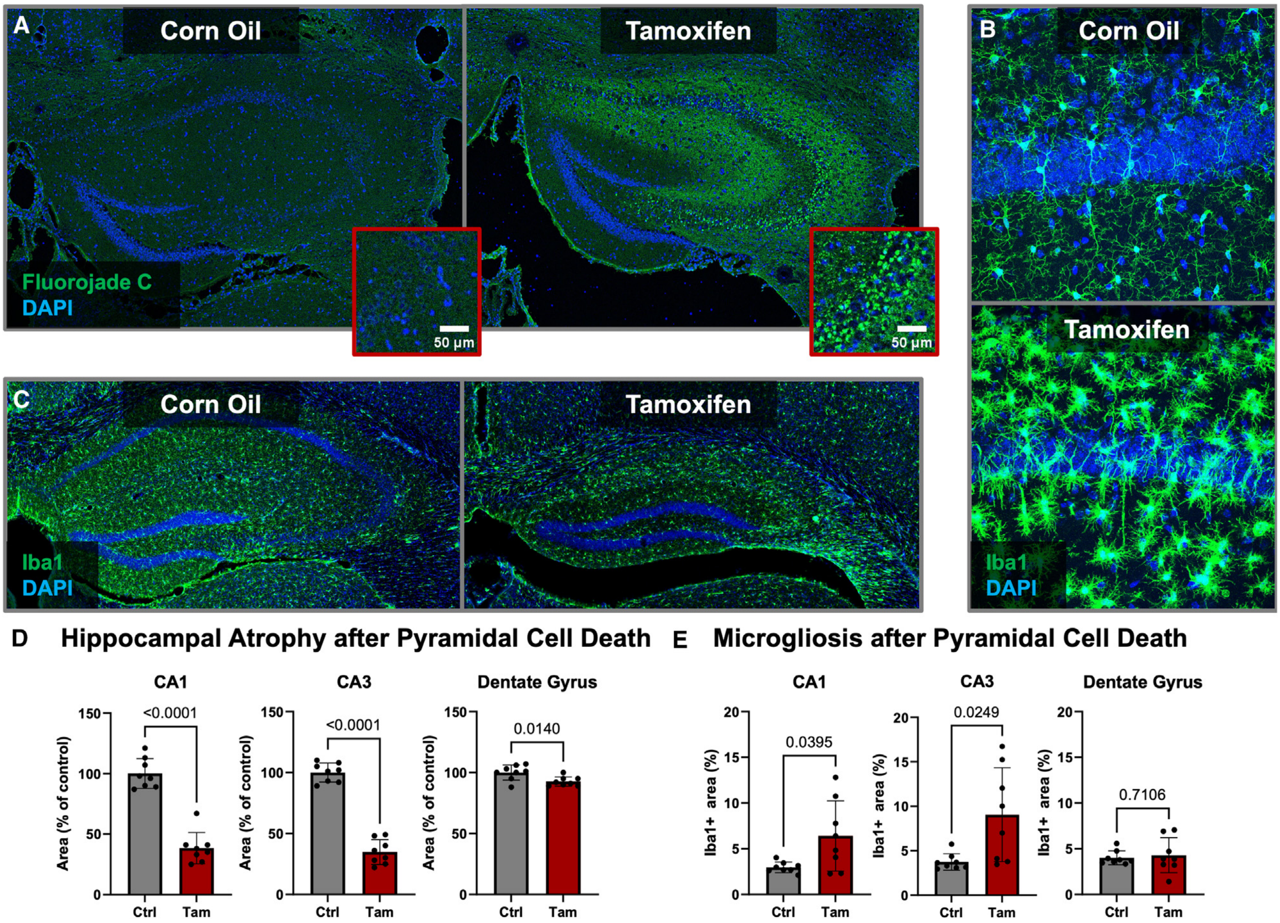

Figure 3. Histopathological consequences of tamoxifen induced DTA expression in hippocampal pyramidal neurons

(A) Fluorojade C and DAPI staining of male diphtheria toxin A (DTA) mice showed prominent neuronal degeneration in the cornu ammonis region 1 week after 3× tamoxifen injections as compared to DTA mice injected with 3× corn oil. Scale bar corresponds to 50 micrometers.

(B) Iba1 and DAPI staining of hippocampal sections from mice presented in (A) show clear microgliosis upon tamoxifen induction.

(C) Microgliosis and prominent hippocampal atrophy in tamoxifen-treated DTA mice that were used to validate the IntelliR pipeline. Eight mice per group (50%) were randomly selected and perfused for histological examination after the IntelliCage based phenotyping.

(D) Quantitative assessment of hippocampal atrophy reveals a prominent shrinkage of the cornu ammonis subregions ($p < 0.001$), in which pyramidal cells have been ablated with tamoxifen, as well as a minor but significant atrophy of the dentate gyrus.

(E) Densitometric analysis of the microglia/macrophage marker Iba1 shows a significant increase in microglia/macrophage density within the CA1 ($p = 0.0395$) and CA3 ($p = 0.0249$) but not the dentate gyrus of tamoxifen-induced DTA mice.

DTA mice were confirmed histologically after testing in the IntelliCages (Figures 3C–3E).

The learning performance of DTA mice was evaluated after induction of hippocampal pyramidal cell death by tamoxifen and compared to healthy corn-oil-treated littermate controls (Figure 4). While control mice showed a significant above-chance performance in all tests ($p < 0.0001$), DTA mice failed to learn clockwise patrolling during the first day and showed significantly slower learning rates than controls ($p < 0.05$) in all tests except place learning and episodic-like memory retrieval. Furthermore, we assessed the performance of mice with respect to individual cognitive components, namely the spatial component using the classical place error (Figure 5A), the contribution of sequential testing (extinction and cognitive flexibility) using the challenge error (Figure 5B) and direction error (Figure 5C), and the temporal component via time error (Figure 5D) and summarized these diverse error types in a weighted cognition index (Figure 5E; Table 1). In comparison to healthy corn-oil controls, tamoxifen-induced DTA mice showed a significantly higher rate of place errors in the first 24 h of both clockwise and counterclockwise patrolling, whereas place and reversal learning, as well as place errors in the episodic-like memory test, were not significantly altered (Figure 5A). Analysis of challenge errors revealed challenge-dependent differences in extinction and cognitive flexibility. While tamoxifen-induced mice made significantly less challenge errors in the multiple reversal learning trial, they showed significantly higher challenge errors in the episodic-like memory reversal acquisition day (Figure 5B). Extinction of the clockwise patrolling task was similar between tamoxifen and corn-oil-treated DTA mice and completed within the first day of counterclockwise

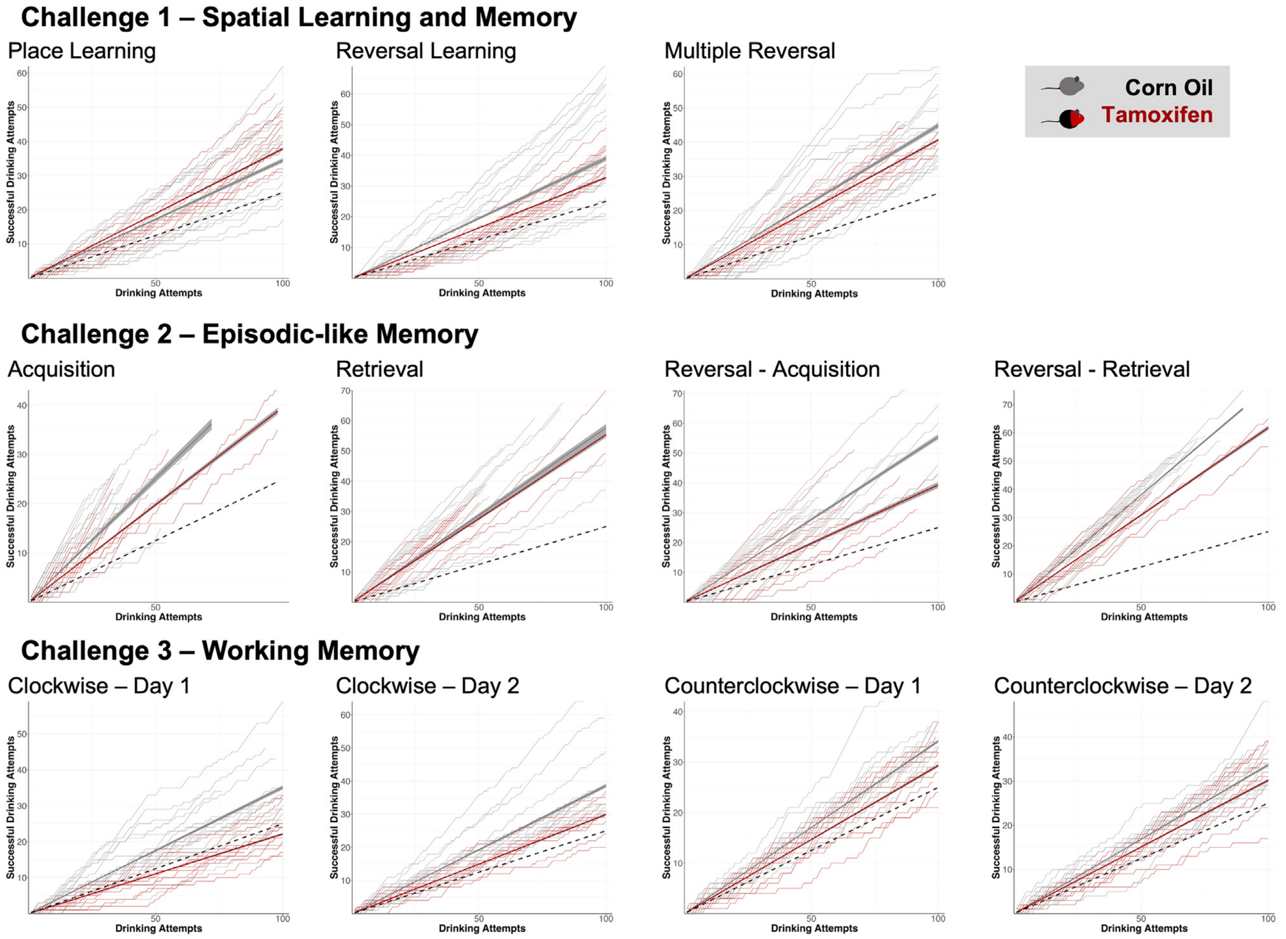

Figure 4. Learning performance of the DTA cohort

To test if the cognitive challenges are sensitive enough to detect hippocampal dysfunction, the learning performance of DTA mice was tested after induction of hippocampal pyramidal cell death by tamoxifen and compared to corn-oil-treated healthy littermate controls. Cumulative successes at each drinking attempt were plotted for individual mice (faint lines) and group performances, calculated by linear regression (bold lines; 95% CI depicted as gray shadows), were compared to the average by chance performance (dashed line) and between groups through estimated marginal means contrasts. While control mice showed a significant above-chance performance in all tests ($p < 0.0001$), DTA mice failed to learn clockwise patrolling during day 1 and showed significantly slower learning rates ($p < 0.05$) than controls in all tests except place learning and episodic-like memory retrieval. Global comparisons were calculated using analysis of covariance (ANCOVA), with specific group comparisons calculated through estimated marginal means contrasts.

patrolling (Figure 5C). Similarly, learning of the temporal component of the episodic-like memory test was comparable between tamoxifen and corn-oil-treated mice (Figure 5D). Taking the different types of errors into account, the cognition index shows that, within each challenge, mice improved their cognitive performance with time (Figure 5E). Furthermore, the cognition index reveals worse cognitive performance in the patrolling task, indicating impaired working memory in mice upon ablation of hippocampal pyramidal neurons (Figure 5E).

Assessing basic mouse behaviors using IntelliR

In addition to the automated cognitive phenotyping, IntelliR provides users with parameters to monitor more basic mouse behavior, such as overall (circadian) activity, exploratory behavior, and repetitive behaviors. These parameters are invaluable for the assessment of the overall health and well-being of mice as they are continuously recorded in a social home cage environment.[2] Analyzing the number of visits of three independent cohorts of healthy female mice during challenge 1 in our IntelliCage paradigm demonstrated that the majority (85.15%) of visits occurred during the dark as compared to the light phase (580.5 ± 156.8 versus 128.3 ± 59.3, $p < 0.001$), highlighting the usefulness of visits as proxy for general activity. Furthermore, an analysis of the number of exploratory visits per test day showed a prominent decrease during the same period (Friedman rank-sum test $p < 0.001$), indicating that exploratory visits are indeed a good proxy of exploratory behavior. While we did not observe differences in general activity, exploratory behavior, or number of repetitive events between tamoxifen and corn-oil-treated DTA mice throughout the challenges (Figures 6A–6C), these parameters can be useful for mouse models with more pronounced motor or stereotypic phenotypes, such as models

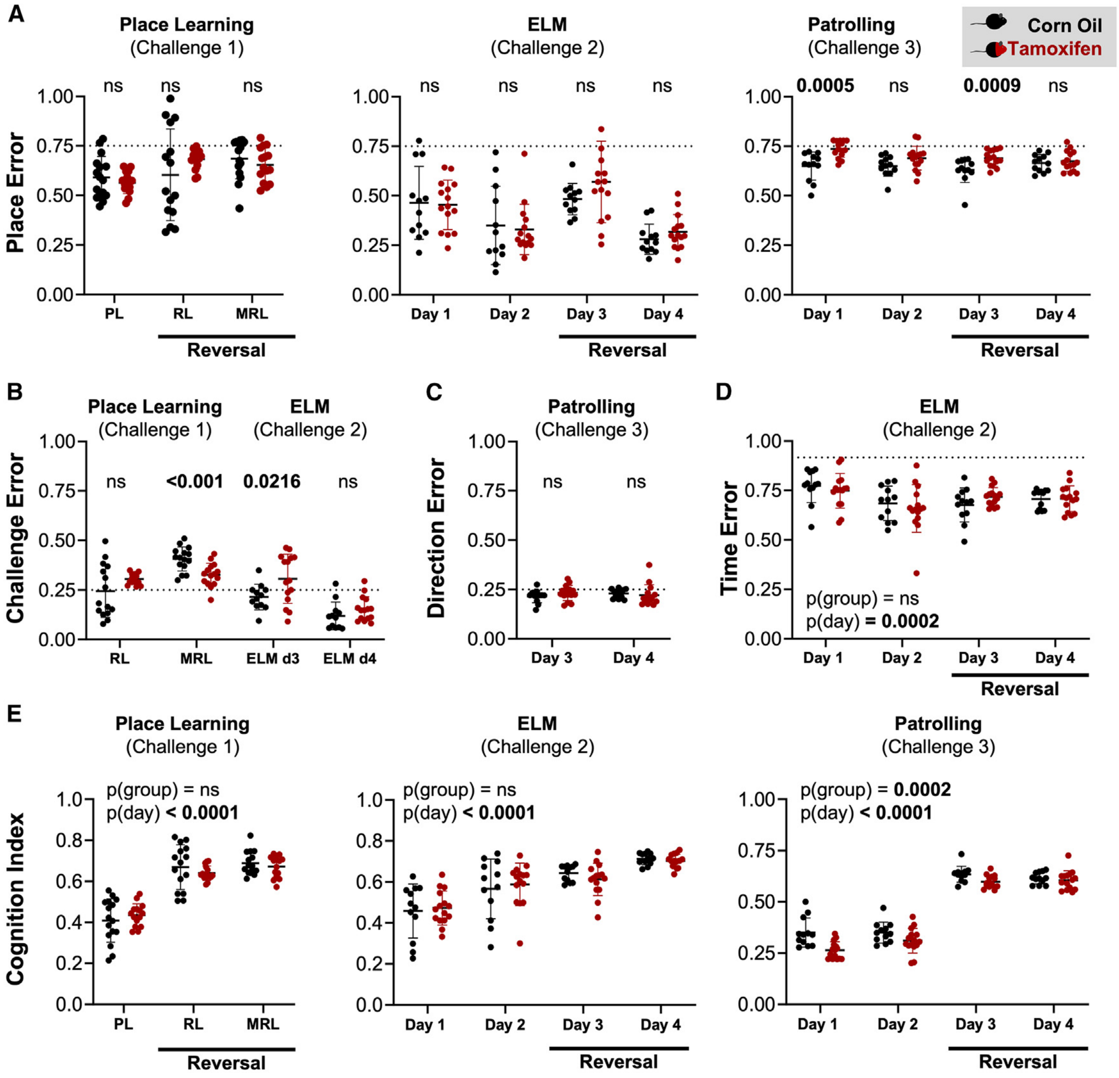

Figure 5. Cognitive performance of tamoxifen-induced (red) versus corn-oil-treated (black) DTA mice

(A) Place errors are plotted to evaluate the spatial component of all cognitive tests. DTA mice show significant deficits during the first day of clockwise ($p = 0.005$) and counterclockwise patrolling ($p = 0.0009$) respectively.

(B and C) Challenge errors (B) and direction errors (C) are presented for all challenges involving a reversal task to evaluate extinction of the previous task and cognitive flexibility. In (B), we show that there was a significantly lower challenge error for the DTA animals during MRL ($p < 0.001$), while their challenge error was significantly higher during ELM day 3 ($p = 0.0216$). The dotted lines indicate the average by-chance performance (3/4 corners are incorrect).

(D) Time errors are plotted to assess learning of the temporal component of the episodic-like memory test. Average by-chance performance is indicated by the dotted line (22/24 h are incorrect). A significant change across days was observed ($p = 0.0002$).

(E) To assess the overall cognitive performance of mice across all challenges, the cognition indices are plotted for all challenges. Taking the different types of errors into account, the cognition index shows that within each challenge, mice significantly improved their cognitive performance with time (all $p < 0.0001$, repeated measures ANOVA for challenge 1 and Friedman's two-way test for challenges 2 and 3). Furthermore, the cognition index shows a significantly worse cognitive performance in the patrolling task in mice upon hippocampal pyramidal cell ablation ($p = 0.0002$, Friedman's two-way test). Data presented as mean ± standard deviation. Pairwise comparisons in (A–D) calculated as described in the method section.

for Huntington's disease, Parkinson's disease, multiple sclerosis, or autism spectrum disorder.[23–26]

DISCUSSION

IntelliR has been designed to offer a comprehensive pipeline for analyzing datasets generated by IntelliCages. As a tool, IntelliCages play a pivotal role in behavioral analysis of mice by enabling independent longitudinal testing in the social context of their home cage. IntelliR standardizes the analysis process, thereby allowing researchers to concentrate more on interpreting their results rather than the intricacies of data analysis.

Furthermore, IntelliR significantly simplifies the process of evaluating basic activity of mice, a crucial metric in assessing their

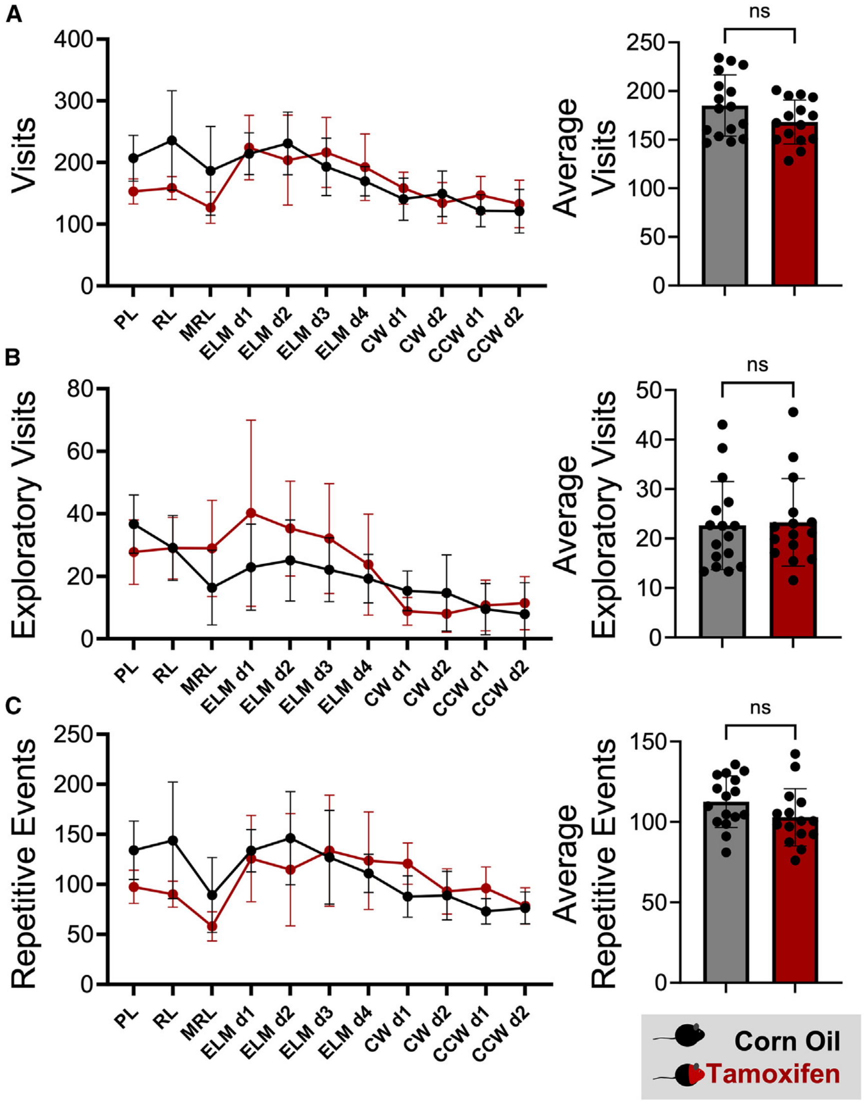

Figure 6. Monitoring basic activity-related mouse behavior using IntelliR

Total number of corner visits (A), exploratory visits (B), and repetitive events (C) per test day (left graphs) and averages over all test days (right graphs) were used to evaluate general activity (A), exploratory behavior (B), and repetitive behavior (C). No differences were observed between tamoxifen-treated (red) and corn-oil-treated (black) DTA mice. Data presented as mean ± standard deviation. Welch's corrected two-sided t tests were used to compare the averaged parameters.

well-being. The primary advantage of the IntelliCage is its ability to monitor and assess mouse behavior with minimum experimenter interference, thereby providing a more familiar and likely less stressful environment for the animals.[2] Another advantage of the IntelliR is that it enables automated cognitive profiling of mice as they are exposed to a diverse set of cognitive challenges and offers a number of additional parameters, which are inaccessible in the IntelliCage Analyzer software.

The selected challenges were based on existing IntelliCage test designs that assess simple place learning and spatial memory,[1] episodic-like memory,[1] as well as reference memory and spatial working memory in a clockwise patrolling task.[10,27,28] For all challenges, reversal tasks were included to evaluate cognitive flexibility in the assessed domains. In addition, we included days without any cognitive task and with unrestricted access to water, which allow the experimenter to clean the cages, assess animal welfare, and appraise extinction of learned behavior.

To independently validate that healthy mice are able to learn these challenges within the allocated testing times, we tested three independent cohorts of female mice and introduced systematic variation to enhance reproducibility and avoid experiment-dependent observations.[29] We demonstrated that our proposed challenges can be successfully learned by healthy mice, with their performance significantly exceeding random chance. Furthermore, we evaluated the sensitivity of these cognitive tasks for measuring hippocampal dysfunction by testing DTA mice after ablation of hippocampal pyramidal neurons. Consistent with previous findings in this model[21] and mice with hippocampal lesions,[9,10] we did not observe differences in the IntelliCage-based place learning and reversal learning tasks. In contrast, the patrolling task revealed notable differences in our DTA model, which is consistent with previous findings in other mouse models of hippocampal dysfunction,[10,27] stressing the importance of this test for the assessment of disturbed behavioral patterns and neuropsychiatric symptoms.

A reliable and frequently used measurement of success in tasks with a spatial component in the IntelliCage is the place error. The place error indicates how often mice fail to visit or nose poke the correct corner, or place preference, which indicates how frequent mice visit or nose poke the rewarded corner.[2,12–15,28,30–39] In contrast to previous studies that calculated place errors based on the number of corner visits or number of nose pokes, IntelliR provides error rates based on the number of drinking attempts. We defined drinking attempts as visits in which mice attempt to access water using the learned operant response (nose poke), thereby minimizing the influence of exploratory corner visits. To evaluate how well mice learn a particular time association in tasks with a temporal component, IntelliR provides a time error. Furthermore, sequential testing of mice in different cognitive tasks requires mice not only to learn the new rule but also to not follow the previous rules.[40,41] To

Table 1. Weights allotted to each type of error when calculating CI for individual challenge days

Challenge	Place Error *(adj.)*	Challenge Error	Time Error	Direction Error
Place learning	100%	–	–	–
Reversal learning	75%	25%	–	–
Multiple reversal learning	75%	25%	–	–
ELM acquisition	75%	–	25%	–
ELM retrieval	75%	–	25%	–
ELM reversal acquisition	50%	25%	25%	–
ELM reversal retrieval	50%	25%	25%	–
Working memory clockwise day 1	100%	–	–	–
Working memory clockwise day 2	100%	–	–	–
Working memory counterclockwise day 1	75%	–	–	25%
Working memory counterclockwise day 2	75%	–	–	25%

ELM, episodic-like memory.

appraise the ability of mice for behavioral extinction, IntelliR provides challenge error and direction error, which indicate how often a mouse follows the rules of the preceding task. To assess the global cognitive performance in all of these components, we introduced the cognition index. Comparing the cognition index of mice across different cognitive tasks revealed that healthy mice improved their cognitive performance with successive tests and outperformed DTA mice in the patrolling task.

As the movement of mice is not directly tracked, the general activity in the IntelliCage is typically measured using the number of corner visits as proxy.[2] Using this approach, a variety of researchers have replicated activity-related phenotypes observed in classical behavioral tests and different automated tracking systems in the IntelliCages.[5,24–26,33,42,43] Furthermore, we recently confirmed that the number of corner visits in the IntelliCage correlates with the locomotor activity in an automated vibration-based phenotyping system.[21] To show that corner visits are indeed a good proxy for activity, we analyzed the number of corner visits of healthy mice during the dark phase as compared to the light phase and found that approximately 85% of the corner visits occur in the active (dark) phase of mice, thereby confirming corner visits as proxy for activity and demonstrating the capacity to monitor circadian rhythms.

To assess exploratory behavior in the IntelliCage, we hypothesized that visits to the conditioning corners, in which mice do not attempt to access water with a nose poke, are due to exploratory behavior and defined these visits as exploratory visits. This hypothesis is based on the assumption that mice quickly learn that access to water is limited to the conditioning corners and requires an operant response, i.e., nose poke. Indeed, learning of the operant response and adaptation to the IntelliCage environment is typically achieved within 1 day, either during an initial habituation or free adaptation phase[11] or directly during a place learning task.[1] As exploratory behavior decreases with increased familiarity to a novel environment such as the IntelliCage,[11,13,28] we assessed the number of exploratory visits of mice during the exposure to the IntelliCage environment and found a significant decrease with time, indicating that exploratory visits can be used as proxy for exploratory behavior.

To assess the intensity and frequency of repetitive behavior, IntelliR provides the number of repetitive nose pokes and the number of repetitive events, both defined as uninterrupted bouts of at least two nose pokes. Although a similar approach has been described previously,[44] the threshold for repetitive nose pokes and repetitive events may need to be adjusted. Careful validation with a mouse model, characterized by consistent repetitive, impulsive, or stereotypic behavior, will help achieve optimal sensitivity and specificity of this readout.

Limitations of the study

This study has limitations that can be broadly categorized into general and technical aspects. The research was conducted using multiple cohorts of healthy mice and one specific model. However, all the subjects were female and of a certain age. Therefore, additional testing is necessary to determine whether the findings are applicable to male mice, mice with different phenotypes, and older mice. Specifically for the last two cases, severe movement limitations, such as hindlimb paralysis in mice with experimental autoimmune encephalomyelitis, can interfere with their ability to successfully enter the conditioning corners. Furthermore, mice that fail to visit or drink in the correct corner, i.e., due to lack of motivation, cognitive impairments, or anxiety, have to be removed from the IntelliCage due to water deprivation. In these cases, the experimenter may consider changing the reward to sucrose solution and providing *ad libitum* access to water outside of the conditioning corners.[2]

A theoretical consideration to keep in mind when using the cognition index is that it assumes that all challenges are learned and that extinction of previously learned tasks requires trial and error, reflected by lower weight of challenge and direction errors as compared to place error. The performance of three independent cohorts of healthy female mice supports this assumption.

The main technical limitation is that the current state of IntelliR requires that all challenges are performed and executed as described by us. These might not be ideal for different experimental settings and questions. We try to mitigate this by providing the design files for the challenges that can be modified. IntelliR is also made available under a GPL-3.0 license, which means its code can be modified as deemed necessary by anyone. It is mainly composed of a set of R-scripts that can be changed for different settings with a moderate amount of knowledge. In our GitHub page (https://github.com/vgastaldi/IntelliR) a tutorial folder is available showing how IntelliR can be modified to work with different behavioral challenges. Additional measurements can be added in the same way.

Alternatively, researchers can consider other dedicated options designed to work with IntelliCage data. Two open-source tools coded in Python, PyMICE[18] and IntelliPy,[45] are available and require different coding level expertise. They differ from

IntelliR regarding their approach to data extraction and how data analysis is conducted. Researchers should evaluate these tools to see if they fulfill their usage needs. Another available option is the FlowR package, a proprietary solution developed by XBehavior/TSE.[10]

In conclusion, we present an analysis pipeline in the form of an R-script, IntelliR, along with a set of challenges designed to provide a sensitive measure for higher cognition profiling. Despite its limitations, our work offers a standardized approach to behavioral valuations, upgrading the usability of IntelliCages and likely contributing to reproducibility in the field.

RESOURCE AVAILABILITY

Lead contact

Further information and requests for resources should be directed to the lead contact, Hannelore Ehrenreich (hannelore.ehrenreich@web.de).

Materials availability

This study did not generate new unique reagents. All materials utilized in this project can be identified and ordered by the information presented.

Data and code availability

- All datasets used in this work are available at the following Zenodo repository: https://doi.org/10.5281/zenodo.14912688
- All code used in this work is available at the following Zenodo repository: https://doi.org/10.5281/zenodo.14912688. Future updates will be available at https://github.com/vgastaldi/IntelliR.
- Any additional information required to reanalyze the data reported in this paper is available from the lead contact upon request.

ACKNOWLEDGMENTS

This work has been funded by the European Research Council (ERC) Advanced Grant to H.E. under the European Union's Horizon Europe research and innovation programme (acronym BREPOCI; grant agreement no. 101054369), as well as an ERC Consolidator Grant to K.W.M. (acronym ALTIBRAIN; grant agreement no. 101043416), in collaboraton with H.E. Furthermore, the study has been fostered by the Max Planck Society and the Max Planck Förderstiftung. Research in the labs of H.E. and K.-A.N. is funded by DFG TRR-274/1 2020-408885537. K.-A.N. is supported by the Adelson Medical Research Foundation. V.D.G. received support from the IMPRS-Genome Science PhD program. For the publication fee, the authors acknowledge financial support from Heidelberg University. The R logo in the graphical abstract (R logo copyright 2016 The R Foundation; https://www.r-project.org/logo/) is used under CC-BY-SA 4.0 (https://creativecommons.org/licenses/by-sa/4.0/). Changes were made to the original logo.

AUTHOR CONTRIBUTIONS

Supervision, H.E. and K.-A.N.; funding acquisition, H.E., K.W.M., and K.-A.N.; concept and design, V.D.G., M.H., J.B.H.W., S.A., and H.E.; data acquisition/generation, V.D.G., M.H., A.R., J.B.H.W., A.-F.W., and A.N.; data analyses/interpretation, V.D.G., M.H., J.B.H.W., A.R., S.K., A.-F.W., A.N., and H.E.; manuscript drafting, V.D.G., M.H., J.B.H.W., and H.E.; display item drafting, V.D.G., M.H., J.B.H.W., and H.E.; continuous critical input and review & editing, M.J.P., L.Y., Y.C., J.-A.C.-S., U.J.B., K.W.M., K.-A.N., and H.E. All authors read and approved the final version of the manuscript.

DECLARATION OF INTERESTS

The authors declare no competing interests.

DECLARATION OF GENERATIVE AI AND AI-ASSISTED TECHNOLOGIES IN THE WRITING PROCESS

During the preparation of this work, the authors used ChatGPT to proofread and enhance readability of parts of the text. ChatGPT was also used to prepare the code for the shiny[46] web interface and debug issues in the analysis pipeline.

STAR★METHODS

Detailed methods are provided in the online version of this paper and include the following:

- KEY RESOURCES TABLE
- EXPERIMENTAL MODEL AND STUDY PARTICIPANT DETAILS
  - Mice
  - Control cohorts
  - DTA cohort
- METHOD DETAILS
  - Transponder placement
  - Model validation
  - IntelliCage experiments
- QUANTIFICATION AND STATISTICAL ANALYSIS
  - IntelliR software
  - Data analysis
  - Statistical analysis

SUPPLEMENTAL INFORMATION

Supplemental information can be found online at https://doi.org/10.1016/j.crmeth.2025.101011.

Received: February 8, 2024
Revised: November 20, 2024
Accepted: February 26, 2025
Published: March 17, 2025

REFERENCES

1. Dere, E., Ronnenberg, A., Tampe, B., Arinrad, S., Schmidt, M., Zeisberg, E., and Ehrenreich, H. (2018). Cognitive, emotional and social phenotyping of mice in an observer-independent setting. Neurobiol. Learn. Mem. *150*, 136–150. https://doi.org/10.1016/j.nlm.2018.02.023.
2. Kiryk, A., Janusz, A., Zglinicki, B., Turkes, E., Knapska, E., Konopka, W., Lipp, H.P., and Kaczmarek, L. (2020). IntelliCage as a tool for measuring mouse behavior - 20 years perspective. Behav. Brain Res. *388*, 112620. https://doi.org/10.1016/j.bbr.2020.112620.
3. Dere, E., Dahm, L., Lu, D., Hammerschmidt, K., Ju, A., Tantra, M., Kästner, A., Chowdhury, K., and Ehrenreich, H. (2014). Heterozygous ambra1 deficiency in mice: a genetic trait with autism-like behavior restricted to the female gender. Front. Behav. Neurosci. *8*, 181. https://doi.org/10.3389/fnbeh.2014.00181.
4. El-Kordi, A., Winkler, D., Hammerschmidt, K., Kästner, A., Krueger, D., Ronnenberg, A., Ritter, C., Jatho, J., Radyushkin, K., Bourgeron, T., et al. (2013). Development of an autism severity score for mice using Nlgn4 null mutants as a construct-valid model of heritable monogenic autism. Behav. Brain Res. *251*, 41–49. https://doi.org/10.1016/j.bbr.2012.11.016.
5. Galsworthy, M.J., Amrein, I., Kuptsov, P.A., Poletaeva, I.I., Zinn, P., Rau, A., Vyssotski, A., and Lipp, H.P. (2005). A comparison of wild-caught wood mice and bank voles in the Intellicage: assessing exploration, daily activity patterns and place learning paradigms. Behav. Brain Res. *157*, 211–217. https://doi.org/10.1016/j.bbr.2004.06.021.
6. Endo, T., Maekawa, F., Võikar, V., Haijima, A., Uemura, Y., Zhang, Y., Miyazaki, W., Suyama, S., Shimazaki, K., Wolfer, D.P., et al. (2011). Automated test of behavioral flexibility in mice using a behavioral sequencing

task in IntelliCage. Behav. Brain Res. *221*, 172–181. https://doi.org/10.1016/j.bbr.2011.02.037.

7. Krackow, S., Vannoni, E., Codita, A., Mohammed, A.H., Cirulli, F., Branchi, I., Alleva, E., Reichelt, A., Willuweit, A., Voikar, V., et al. (2010). Consistent behavioral phenotype differences between inbred mouse strains in the IntelliCage. Genes Brain Behav. *9*, 722–731. https://doi.org/10.1111/j.1601-183X.2010.00606.x.
8. Arinrad, S., Wilke, J.B.H., Seelbach, A., Doeren, J., Hindermann, M., Butt, U.J., Steixner-Kumar, A.A., Spieth, L., Ronnenberg, A., Pan, H., et al. (2022). NMDAR1 autoantibodies amplify behavioral phenotypes of genetic white matter inflammation: a mild encephalitis model with neuropsychiatric relevance. Mol. Psychiatry *27*, 4974–4983. https://doi.org/10.1038/s41380-021-01392-8.
9. Voikar, V., Colacicco, G., Gruber, O., Vannoni, E., Lipp, H.P., and Wolfer, D.P. (2010). Conditioned response suppression in the IntelliCage: assessment of mouse strain differences and effects of hippocampal and striatal lesions on acquisition and retention of memory. Behav. Brain Res. *213*, 304–312. https://doi.org/10.1016/j.bbr.2010.05.019.
10. Voikar, V., Krackow, S., Lipp, H.P., Rau, A., Colacicco, G., and Wolfer, D.P. (2018). Automated dissection of permanent effects of hippocampal or prefrontal lesions on performance at spatial, working memory and circadian timing tasks of C57BL/6 mice in IntelliCage. Behav. Brain Res. *352*, 8–22. https://doi.org/10.1016/j.bbr.2017.08.048.
11. Vannoni, E., Voikar, V., Colacicco, G., Sánchez, M.A., Lipp, H.P., and Wolfer, D.P. (2014). Spontaneous behavior in the social homecage discriminates strains, lesions and mutations in mice. J. Neurosci. Methods *234*, 26–37. https://doi.org/10.1016/j.jneumeth.2014.04.026.
12. Berry, A., Amrein, I., Nötzli, S., Lazic, S.E., Bellisario, V., Giorgio, M., Pelicci, P.G., Alleva, E., Lipp, H.P., and Cirulli, F. (2012). Sustained hippocampal neurogenesis in females is amplified in P66(Shc-/-) mice: An animal model of healthy aging. Hippocampus *22*, 2249–2259. https://doi.org/10.1002/hipo.22042.
13. Kalm, M., Karlsson, N., Nilsson, M.K.L., and Blomgren, K. (2013). Loss of hippocampal neurogenesis, increased novelty-induced activity, decreased home cage activity, and impaired reversal learning one year after irradiation of the young mouse brain. Exp. Neurol. *247*, 402–409. https://doi.org/10.1016/j.expneurol.2013.01.006.
14. Huo, K., Sun, Y., Li, H., Du, X., Wang, X., Karlsson, N., Zhu, C., and Blomgren, K. (2012). Lithium reduced neural progenitor apoptosis in the hippocampus and ameliorated functional deficits after irradiation to the immature mouse brain. Mol. Cell. Neurosci. *51*, 32–42. https://doi.org/10.1016/j.mcn.2012.07.002.
15. Roughton, K., Kalm, M., and Blomgren, K. (2012). Sex-dependent differences in behavior and hippocampal neurogenesis after irradiation to the young mouse brain. Eur. J. Neurosci. *36*, 2763–2772. https://doi.org/10.1111/j.1460-9568.2012.08197.x.
16. van Dijk, R.M., Lazic, S.E., Slomianka, L., Wolfer, D.P., and Amrein, I. (2016). Large-scale phenotyping links adult hippocampal neurogenesis to the reaction to novelty. Hippocampus *26*, 646–657. https://doi.org/10.1002/hipo.22548.
17. Puscian, A., Leski, S., Gorkiewicz, T., Meyza, K., Lipp, H.P., and Knapska, E. (2014). A novel automated behavioral test battery assessing cognitive rigidity in two genetic mouse models of autism. Front. Behav. Neurosci. *8*, 140. https://doi.org/10.3389/fnbeh.2014.00140.
18. Dzik, J.M., Puścian, A., Mijakowska, Z., Radwanska, K., and Łęski, S. (2018). PyMICE: APython library for analysis of IntelliCage data. Behav. Res. Methods *50*, 804–815. https://doi.org/10.3758/s13428-017-0907-5.
19. Agarwal, A., Dibaj, P., Kassmann, C.M., Goebbels, S., Nave, K.A., and Schwab, M.H. (2012). In vivo imaging and noninvasive ablation of pyramidal neurons in adult NEX-CreERT2 mice. Cereb. Cortex *22*, 1473–1486. https://doi.org/10.1093/cercor/bhr214.
20. Wilke, J.B.H., Hindermann, M., Moussavi, A., Butt, U.J., Dadarwal, R., Berghoff, S.A., Sarcheshmeh, A.K., Ronnenberg, A., Zihsler, S., Arinrad, S., et al. (2021). Inducing sterile pyramidal neuronal death in mice to model distinct aspects of gray matter encephalitis. Acta Neuropathol. Commun. *9*, 121. https://doi.org/10.1186/s40478-021-01214-6.
21. Wilke, J.B.H., Hindermann, M., Berghoff, S.A., Zihsler, S., Arinrad, S., Ronnenberg, A., Barnkothe, N., Steixner-Kumar, A.A., Röglin, S., Stöcker, W., et al. (2021). Autoantibodies against NMDA receptor 1 modify rather than cause encephalitis. Mol. Psychiatry *26*, 7746–7759. https://doi.org/10.1038/s41380-021-01238-3.
22. Lipp, H.P., Krackow, S., Turkes, E., Benner, S., Endo, T., and Russig, H. (2023). IntelliCage: the development and perspectives of a mouse- and user-friendly automated behavioral test system. Front. Behav. Neurosci. *17*, 1270538. https://doi.org/10.3389/fnbeh.2023.1270538.
23. Balci, F., Oakeshott, S., Shamy, J.L., El-Khodor, B.F., Filippov, I., Mushlin, R., Port, R., Connor, D., Paintdakhi, A., Menalled, L., et al. (2013). High-Throughput Automated Phenotyping of Two Genetic Mouse Models of Huntington's Disease. PLoS Curr. https://doi.org/10.1371/currents.hd.124aa0d16753f88215776fba102ceb29.
24. Menalled, L.B., Kudwa, A.E., Oakeshott, S., Farrar, A., Paterson, N., Filippov, I., Miller, S., Kwan, M., Olsen, M., Beltran, J., et al. (2014). Genetic deletion of transglutaminase 2 does not rescue the phenotypic deficits observed in R6/2 and zQ175 mouse models of Huntington's disease. PLoS One *9*, e99520. https://doi.org/10.1371/journal.pone.0099520.
25. Oakeshott, S., Balci, F., Filippov, I., Murphy, C., Port, R., Connor, D., Paintdakhi, A., Lesauter, J., Menalled, L., Ramboz, S., et al. (2011). Circadian Abnormalities in Motor Activity in a BAC Transgenic Mouse Model of Huntington's Disease. PLoS Curr. *3*, RRN1225. https://doi.org/10.1371/currents.RRN1225.
26. Rudenko, O., Tkach, V., Berezin, V., and Bock, E. (2009). Detection of early behavioral markers of Huntington's disease in R6/2 mice employing an automated social home cage. Behav. Brain Res. *203*, 188–199. https://doi.org/10.1016/j.bbr.2009.04.034.
27. Weyer, S.W., Klevanski, M., Delekate, A., Voikar, V., Aydin, D., Hick, M., Filippov, M., Drost, N., Schaller, K.L., Saar, M., et al. (2011). APP and APLP2 are essential at PNS and CNS synapses for transmission, spatial learning and LTP. EMBO J. *30*, 2266–2280. https://doi.org/10.1038/emboj.2011.119.
28. Onishchenko, N., Tamm, C., Vahter, M., Hökfelt, T., Johnson, J.A., Johnson, D.A., and Ceccatelli, S. (2007). Developmental exposure to methylmercury alters learning and induces depression-like behavior in male mice. Toxicol. Sci. *97*, 428–437. https://doi.org/10.1093/toxsci/kfl199.
29. Richter, S.H., Garner, J.P., Auer, C., Kunert, J., and Würbel, H. (2010). Systematic variation improves reproducibility of animal experiments. Nat. Methods *7*, 167–168. https://doi.org/10.1038/nmeth0310-167.
30. Kiryk, A., Aida, T., Tanaka, K., Banerjee, P., Wilczynski, G.M., Meyza, K., Knapska, E., Filipkowski, R.K., Kaczmarek, L., and Danysz, W. (2008). Behavioral characterization of GLT1 (+/-) mice as a model of mild glutamatergic hyperfunction. Neurotox. Res. *13*, 19–30. https://doi.org/10.1007/BF03033364.
31. Codita, A., Gumucio, A., Lannfelt, L., Gellerfors, P., Winblad, B., Mohammed, A.H., and Nilsson, L.N.G. (2010). Impaired behavior of female tg-ArcSwe APP mice in the IntelliCage: A longitudinal study. Behav. Brain Res. *215*, 83–94. https://doi.org/10.1016/j.bbr.2010.06.034.
32. Codita, A., Mohammed, A.H., Willuweit, A., Reichelt, A., Alleva, E., Branchi, I., Cirulli, F., Colacicco, G., Voikar, V., Wolfer, D.P., et al. (2012). Effects of spatial and cognitive enrichment on activity pattern and learning performance in three strains of mice in the IntelliMaze. Behav. Genet. *42*, 449–460. https://doi.org/10.1007/s10519-011-9512-z.
33. Robinson, L., and Riedel, G. (2014). Comparison of automated home-cage monitoring systems: emphasis on feeding behaviour, activity and spatial learning following pharmacological interventions. J. Neurosci. Methods *234*, 13–25. https://doi.org/10.1016/j.jneumeth.2014.06.013.
34. Konopka, W., Kiryk, A., Novak, M., Herwerth, M., Parkitna, J.R., Wawrzyniak, M., Kowarsch, A., Michaluk, P., Dzwonek, J., Arnsperger, T., et al. (2010). MicroRNA loss enhances learning and memory in mice.

J. Neurosci. *30*, 14835–14842. https://doi.org/10.1523/JNEUROSCI.3030-10.2010.

35. Jaholkowski, P., Kiryk, A., Jedynak, P., Ben Abdallah, N.M., Knapska, E., Kowalczyk, A., Piechal, A., Blecharz-Klin, K., Figiel, I., Lioudyno, V., et al. (2009). New hippocampal neurons are not obligatory for memory formation; cyclin D2 knockout mice with no adult brain neurogenesis show learning. Learn. Mem. *16*, 439–451. https://doi.org/10.1101/lm.1459709.
36. Barlind, A., Karlsson, N., Björk-Eriksson, T., Isgaard, J., and Blomgren, K. (2010). Decreased cytogenesis in the granule cell layer of the hippocampus and impaired place learning after irradiation of the young mouse brain evaluated using the IntelliCage platform. Exp. Brain Res. *201*, 781–787. https://doi.org/10.1007/s00221-009-2095-8.
37. Faizi, M., Bader, P.L., Tun, C., Encarnacion, A., Kleschevnikov, A., Belichenko, P., Saw, N., Priestley, M., Tsien, R.W., Mobley, W.C., and Shamloo, M. (2011). Comprehensive behavioral phenotyping of Ts65Dn mouse model of Down syndrome: activation of beta1-adrenergic receptor by xamoterol as a potential cognitive enhancer. Neurobiol. Dis. *43*, 397–413. https://doi.org/10.1016/j.nbd.2011.04.011.
38. Kiryk, A., Mochol, G., Filipkowski, R.K., Wawrzyniak, M., Lioudyno, V., Knapska, E., Gorkiewicz, T., Balcerzyk, M., Leski, S., Leuven, F.V., et al. (2011). Cognitive abilities of Alzheimer's disease transgenic mice are modulated by social context and circadian rhythm. Curr. Alzheimer Res. *8*, 883–892. https://doi.org/10.2174/156720511798192745.
39. Mechan, A.O., Wyss, A., Rieger, H., and Mohajeri, M.H. (2009). A comparison of learning and memory characteristics of young and middle-aged wild-type mice in the IntelliCage. J. Neurosci. Methods *180*, 43–51. https://doi.org/10.1016/j.jneumeth.2009.02.018.
40. Uengoer, M., Lissek, S., Tegenthoff, M., Manahan-Vaughan, D., and Lachnit, H. (2020). Principles of extinction learning of nonaversive experience. Neuroform *26*, 151–159. https://doi.org/10.1515/nf-2020-0013.
41. Bouton, M.E., Maren, S., and McNally, G.P. (2021). BEHAVIORAL AND NEUROBIOLOGICAL MECHANISMS OF PAVLOVIAN AND INSTRUMENTAL EXTINCTION LEARNING. Physiol. Rev. *101*, 611–681. https://doi.org/10.1152/physrev.00016.2020.
42. Kulesskaya, N., Võikar, V., Peltola, M., Yegutkin, G.G., Salmi, M., Jalkanen, S., and Rauvala, H. (2013). CD73 is a major regulator of adenosinergic signalling in mouse brain. PLoS One *8*, e66896. https://doi.org/10.1371/journal.pone.0066896.
43. Jedynak, P., Jaholkowski, P., Wozniak, G., Sandi, C., Kaczmarek, L., and Filipkowski, R.K. (2012). Lack of cyclin D2 impairing adult brain neurogenesis alters hippocampal-dependent behavioral tasks without reducing learning ability. Behav. Brain Res. *227*, 159–166. https://doi.org/10.1016/j.bbr.2011.11.007.
44. Endo, T., Kakeyama, M., Uemura, Y., Haijima, A., Okuno, H., Bito, H., and Tohyama, C. (2012). Executive function deficits and social-behavioral abnormality in mice exposed to a low dose of dioxin in utero and via lactation. PLoS One *7*, e50741. https://doi.org/10.1371/journal.pone.0050741.
45. Ruffini, N., Müller, M.B., Schmitt, U., and Gerber, S. (2021). IntelliPy: a GUI for analyzing IntelliCage data. Bioinformatics *37*, 3972–3973. https://doi.org/10.1093/bioinformatics/btab682.
46. Chang, W., Cheng, J., Allaire, J.J., Sievert, C., Schloerke, B., Xie, Y., Allen, J., McPherson, J., Dipert, A., and Borges, B. (2023). shiny: Web Application Framework for R. https://CRAN.R-project.org/package=shiny.
47. Wickham, H. (2016). ggplot2: Elegant Graphics for Data Analysis (Springer-Verlag).
48. Fox, J., and Weisberg, S. (2019). An R Companion to Applied Regression, Third Edition (Sage).
49. Ben-Shachar, M., Lüdecke, D., and Makowski, D. (2020). effectsize: Estimation of Effect Size Indices and Standardized Parameters. J. Open Source Softw. *5*, 2815.
50. Kassambara, A. (2023). rstatix: Pipe-Friendly Framework for Basic Statistical Tests. https://CRAN.R-project.org/package=rstatix.
51. Dinno, A. (2017). dunn.test: Dunn?s Test of Multiple Comparisons Using Rank Sums. https://CRAN.R-project.org/package=dunn.test.
52. Hothorn, T., Hornik, K., Wiel, M.A.v.d., and Zeileis, A. (2008). Implementing a class of permutation tests: The coin package. J. Stat. Softw. *28*, 1–23.
53. Hothorn, T., Bretz, F., and Westfall, P. (2008). Simultaneous Inference in General Parametric Models. Biom. J. *50*, 346–363.
54. Lenth, R.V. (2023). emmeans: Estimated Marginal Means, aka Least-Squares Means. https://CRAN.R-project.org/package=emmeans.
55. Schindelin, J., Arganda-Carreras, I., Frise, E., Kaynig, V., Longair, M., Pietzsch, T., Preibisch, S., Rueden, C., Saalfeld, S., Schmid, B., et al. (2012). Fiji: an open-source platform for biological-image analysis. Nat. Methods *9*, 676–682. https://doi.org/10.1038/nmeth.2019.
56. Lipina, T.V., and Roder, J.C. (2013). Co-learning facilitates memory in mice: A new avenue in social neuroscience. Neuropharmacology *64*, 283–293. https://doi.org/10.1016/j.neuropharm.2012.06.054.
57. Lappe-Siefke, C., Goebbels, S., Gravel, M., Nicksch, E., Lee, J., Braun, P.E., Griffiths, I.R., and Nave, K.A. (2003). Disruption of Cnp1 uncouples oligodendroglial functions in axonal support and myelination. Nat. Genet. *33*, 366–374. https://doi.org/10.1038/ng1095.
58. Ivanova, A., Signore, M., Caro, N., Greene, N.D.E., Copp, A.J., and Martinez-Barbera, J.P. (2005). In vivo genetic ablation by Cre-mediated expression of diphtheria toxin fragment A. Genesis *43*, 129–135. https://doi.org/10.1002/gene.20162.
59. Dere, E., Dere, D., de Souza Silva, M.A., Huston, J.P., and Zlomuzica, A. (2018). Fellow travellers: Working memory and mental time travel in rodents. Behav. Brain Res. *352*, 2–7. https://doi.org/10.1016/j.bbr.2017.03.026.
60. Binder, S., Dere, E., and Zlomuzica, A. (2015). A critical appraisal of the what-where-when episodic-like memory test in rodents: Achievements, caveats and future directions. Prog. Neurobiol. *130*, 71–85. https://doi.org/10.1016/j.pneurobio.2015.04.002.
61. R Core Team (2023). R: A Language and Environment for Statistical Computing (R Foundation for Statistical Computing).
62. Tomczak, M., and Tomczak, E. (2014). The need to report effect size estimates revisited. Trends in Sport Sciences *1*, 19–25. https://doi.org/10.13140/2.1.4929.7923.

STAR★METHODS

KEY RESOURCES TABLE

REAGENT or RESOURCE	SOURCE	IDENTIFIER
Antibodies		
Iba1	Wako	RRID:AB_839504
Chemicals, peptides, and recombinant proteins		
Tamoxifen	Sigma-Aldrich	T5648
Corn oil	Sigma-Aldrich	C8267
DAPI	Sigma-Aldrich	D9542
Fluorojade C	Sigma-Aldrich	AG325
2,2,2-tribromoethanol	Sigma-Aldrich	T48402
Deposited data		
All datasets used in the work and a data dictionary with all calculated variables	This study	https://doi.org/10.5281/zenodo.14912688
Experimental models: Organisms/strains		
Double heterozygous NexCreERT2 x Rosa26-eGFP-DTA mice	Max Planck Institute for Multidisciplinary Sciences	Neurod6$^{tm2.1(cre/ERT2)Kan}$, Gt(ROSA)26$^{Sortm1(DTA)Jpmb}$
Heterozygous NexCreERT2 mice	Max Planck Institute for Multidisciplinary Sciences	Neurod6$^{tm2.1(cre/ERT2)Kan}$
Heterozygous CNP-Cre mice	Max Planck Institute for Multidisciplinary Sciences	Cnp$^{tm1(cre)Kan}$
Wildtype mice	Max Planck Institute for Multidisciplinary Sciences	C57B6
Software and algorithms		
IntelliR	This study	https://doi.org/10.5281/zenodo.14912688 and https://github.com/vgastaldi/IntelliR
R 4.3.0	R Foundation	; RRID:SCR_001905
shiny v1.7.5	Chang et al.[46]	https://shiny.posit.co/; RRID:SCR_001626
ggplot2 3.4.3	Wickham[47]	https://ggplot2.tidyverse.org/; RRID:SCR_014601
car 3.1–2	Fox and Weisberg[48]	https://r-forge.r-project.org/projects/car/; RRID:SCR_022137
effectsize 0.8.6	Ben-Shachar et al.[49]	https://easystats.github.io/effectsize/
rstatix 0.7.2	Kassambara[50]	https://rpkgs.datanovia.com/rstatix/; RRID:SCR_021240
dunn.test 1.3.5	Dinno[51]	https://CRAN.R-project.org/package=dunn.test
coin 1.4–2	Hothorn et al.[52]	http://coin.r-forge.r-project.org/
multcomp 1.4–25	Hothorn et al.[53]	http://multcomp.r-forge.r-project.org/; RRID:SCR_018255
emmeans v1.8.8	Lenth[54]	https://rvlenth.github.io/emmeans/; RRID:SCR_018734
FIJI/ImageJ	Schindelin[55]	https://imagej.net/software/fiji/; RRID:SCR_002285
Other		
IntelliCage	TSE Systems	RRID:SCR_017404
RFID Transponders	TSE Systems	PM162-8

EXPERIMENTAL MODEL AND STUDY PARTICIPANT DETAILS

Mice

All animal experiments were approved by the local animal care and use committee (LAVES, Niedersaechsisches Landesamt für Verbraucherschutz und Lebensmittelsicherheit, Oldenburg, Germany, license number 33.19-42502-04-18/2803) in accordance with the German animal protection law. Mice were housed in groups of up to 16 in a temperature (~22°C) and humidity (~50%) controlled environment, 12h light/dark cycle with food (standard food, Sniff Spezialdiäten, Germany) and water *ad libitum*. Cages were equipped with wood-chip bedding end nesting material (Sizzle Nest, Datesand, United Kingdom). Experimental mice

were weaned at postnatal day 21 and separated by sex and genotype to avoid co-learning, inclusion effects, or aggressive behavior against possibly affected animals.[1,2,56] Investigators performing animal experiments were unaware of group assignment ('fully blinded').

Control cohorts

To avoid experiment-specific observations and to increase systematic variation to improve reproducibility,[29] we analyzed data from 3 cohorts of female C57BL/6 mice aged 7–13 months with the following genotypes: heterozygous CNP-Cre[57] (*n* = 8), heterozygous NexCreERT2[19] (*n* = 16), and wildtype (*n* = 15).

DTA cohort

Female C57BL/6 mice double heterozygous for Neurod6$^{tm2.1(cre/ERT2)Kan}$ ('NexCreERT2',[19]) and Gt(ROSA)26Sor$^{tm1(DTA)Jpmb}$ ('Rosa26-eGFP-DTA',[58]) (*n* = 13), a tamoxifen inducible diphtheria toxin chain A allele, were used at the age of 7–8 months. Pyramidal cells were ablated as previously described[20] via intraperitoneal injections of 100mg tamoxifen (CAS#10540-29-1 T5648, Sigma-Aldrich) per kilogram body weight per day using a stock solution of 10 mg/mL tamoxifen in corn oil (C8267, Sigma-Aldrich) for 3 consecutive days. Control littermates received 10mL corn oil per kilogram body weight per day for 3 consecutive days. Place learning was started one week after the first tamoxifen injection. At this time point, pronounced pyramidal cell death and microgliosis were observed in the *cornu ammonis* region of male DTA littermates (*n* = 4) after the same tamoxifen treatment.

METHOD DETAILS

Transponder placement

Mice were anesthetized with Avertin (2,2,2-tribromoethanol, Sigma-Aldrich, Taufkirchen, Germany, in ddH$_2$O i.p. 300 mg/kg body weight). After a small incision (2–3 mm) of the neck skin, one ISO standard transponder (12mm length, 2mm diameter, PM162-8; TSE Systems, Berlin, Germany) per mouse was implanted below the skin, enabling the IntelliCage system to individually identify each mouse. After closing the incision with suture and covering the eyes with protection salve, mice were put in their home cage, which was placed on a heating plate (~38°C) to avoid hypothermia during the wake-up phase.

Model validation

After behavioral phenotyping, hippocampal atrophy and microgliosis were confirmed histologically in tamoxifen- and corn oil-treated control DTA mice (*n* = 8 per group) as previously described.[20,21] Imaging parameters were kept constant during image acquisition. Image processing and segmentation of hippocampal regions were performed manually by a blinded investigator using FIJI/ImageJ.[55] Densitometric analysis of Iba1 was performed using uniform thresholding. Per mouse, 3–4 hippocampi from two 30μm serial coronal sections at bregma level 1.34–1.44mm and 1.64–1.74mm were analyzed.

IntelliCage experiments

The structure and function of the IntelliCage paradigm has been previously described in detail.[1,2] In short, this setup allows observer-independent measurement of rodent behavior within a social environment. Being set inside a standard type IV rodent cage (20.5 × 55 × 38.5cm, Techniplast), the IntelliCage consists of 4 right-angled triangular conditioning chambers ("corners", 15 × 15 × 21 cm, Figure 1A) and a food hopper with *ad libitum* access. The system provides 3 basic readouts for each conditioning chamber (Figure 1B): visits (ring radiofrequency identification antenna within the tube for recognizing individual mice via detection and readout of the implanted transponder), nose pokes (mouse disrupts light barriers in front of doors) and licks (number of contacts with lickometer). The IntelliCage system includes software to design experiments (when and where do animals get access to drinking nipples), online monitoring and collection of data. Using this software, we designed 3 challenges to test different cognitive abilities of mice. In between the challenges, mice received free access to water in all corners for 24h. During these extinction days, the health status and body weights of mice were assessed and IntelliCages were cleaned. Mice were excluded from the IntelliCage experiment, if they performed <100 licks/day on 2 consecutive days, if their health status deteriorated or if their body weight dropped.

Challenge 1 – Spatial Learning and Memory (Figure 1C): Mice were placed into the IntelliCage with free access to all water bottles when performing a nose poke (habituation) during the first light (6a.m.-6p.m.) and dark phase (6p.m.-6a.m.). Starting with the second light phase, water access was restricted to the active phase (dark phase, 12h) of the animals. Based on their transponder IDs, mice were divided into 4 groups per cage. During the second dark phase, each group was assigned to a single corner, where a nose poke would lead to water access. In the other 3 corners, doors would not open after performing a nose poke (*place learning*). In the third dark phase, each group was allocated the corner diagonally opposite of the previously correct corner (*reversal learning*). During the fourth dark phase, the correct corner for each group switched every 3 h between the 2 formerly correct ones (*multiple reversal learning*).

Challenge 2 - Episodic-like Memory (ELM, Figure 1D): This challenge is based on the episodic-like memory test developed by Dere and colleagues[1] and was modified to include a reversal of the where (corner) and when (time) component. For a detailed description

of this task and the assessment of episodic-like memory in rodents, we kindly refer to the original method paper and 2 excellent reviews by Dere and colleagues.[1,59,60] In brief, the cognitive performance of test mice in this challenge depends on their individual recollection of contextual memory in regard to the factors of what (access to water), where (which corner), and when (time window). Briefly, the challenge starts with access to water in all corners after performing a nose poke. During the second dark phase, each group was allotted to the same corner that was rewarded during the place learning task. Water access was only granted between 10p.m. and 12p.m. (ELM – acquisition). On the following dark phase, the same corner was rewarded with water access during the same time window to retrieve the previously learned task (ELM – retrieval). On the following 2 dark phases, the correct corner was again changed to the one diagonally opposite of the previously rewarded corner and the time window was shifted forward to 8-10p.m. (ELM reversal – acquisition and retrieval). The second time window for ELM reversal was specifically chosen to be set before the first one to avoid mice trying to get water access by extending their attempts into a new time window afterward. Therefore, this task had to be learned anew and could not be fulfilled simply based on the first ELM task.

Challenge 3 – Working Memory (Figure 1E): This challenge was programmed to change the correct corner after each correct nose poke in a clockwise pattern for 48h (*patrolling day 1 & 2*), followed by a switch to a counterclockwise pattern for the following 48h (*patrolling day 3 & 4*). The challenge starts with access to water in all corners after performing a nose poke during the first light and dark phase. To increase motivation and keep the start of cognitive testing consistent between challenges, mice were water deprived for 12h during the second light phase. After the beginning of the patrolling task in the second dark phase, all corners would give access to water if a nose poke is performed. The first nose poke of each individual mouse allocated this corner as the starting point for this challenge and therefore activated the patrolling protocol. After the experiment was stopped, the animals were transferred back to their home cages with food and water *ad libitum*.

QUANTIFICATION AND STATISTICAL ANALYSIS

IntelliR software

The present work introduces IntelliR, an easy-to-use platform for extracting relevant variables from the challenges described above via an R-script.[61] It takes the form of a shiny-based[46] web interface, where users enter basic details from their experiment and receive processed variables, along with an initial statistical assessment and ggplot2[47] figures that can be used for discussions. A full description of the required information and file organization, including tested package versions, is available in the provided documentation in the GitHub page https://github.com/vgastaldi/IntelliR. The dataset for the DTA experiment and a data dictionary with all calculated variables are also provided there.

Data analysis

In addition to the basic measurements described in the IntelliCage Experiments section (i.e., visits, nose pokes, licks), IntelliR provides 9 main additional measurements calculated for the previously outlined challenges. However, the basic R script can be used and adapted to any IntelliCage design after minimal changes.

The first pair of measurements evaluates the purpose of the visit executed by the mice. Exploratory visits are visits to corners where the mouse does not do a nose poke, in contrast to drinking attempts, which are visits where at least one nose poke was done. If more than one nose poke is performed in a single visit to a corner, it is possible that enhanced impulsivity with repetitive behavior has occurred. To quantify the frequency and intensity of potentially repetitive behaviors, the software extracts both the number of repetitive events, defined by the number of visits with multiple nose pokes (repetitive behavior frequency), as well as the number of repeated nose pokes (repetitive behavior total) as proxy of intensity. Of note, the repetitive behavior total can be triggered more than once in a single visit if, after a lick is registered, the mouse continues to make nose pokes without further licks. For example, 10 uninterrupted nose pokes in a single visit would result in a repetitive behavior frequency of 1 and a repetitive behavior total of 9.

The other 5 measurements are directly connected to the challenges and are named place error (PE), challenge error (CE), time error (TE), direction error (DE), and cognition index (CI). The first 4 reflect the specifics of the challenges, while CI compiles them to assess the performance of the mice. It is important to highlight that no single challenge contains all errors. The most basic error calculated is PE, which represents the ratio of drinking attempts made by a mouse in the incorrect corner in relation to all drinking attempts made. It is calculated for all challenges using the following formula:

$$PE = \frac{\textit{Drinking attempts at incorrect corners}}{\textit{Total drinking attempts}}$$

After the first day of each challenge, mice may remember the previously rewarded corner and be particularly drawn to it. CE is calculated in a similar fashion to PE, but takes into account whether the corner was rewarded in the previous challenge immediately before. It is calculated for Reversal Learning, Multiple Reversal Learning, Episodic-like Memory 2 Acquisition, and Episodic-like Memory 2 Retrieval. For challenges where CE is calculated, the formula for CE is:

$$CE = \frac{\textit{Drinking attempts at previously correct corner}}{\textit{Total drinking attempts}}$$

Challenge 2 integrates a time component that is also evaluated by IntelliR. TE is calculated for all 4 days and represents the proportion of drinking attempts made outside of the allotted time window. The formula for TE is:

$$TE = \frac{\textit{Drinking attempts outside of the time window}}{\textit{Total drinking attempts}}$$

The last error, DE, is exclusive to days 3 and 4 of Challenge 3. On days 1 and 2, mice had to move clockwise to be able to continue drinking. On days 3 and 4, the movement changes to counterclockwise. DE evaluates how many times the animals go to the corner directly clockwise of the most recently rewarded corner. For example, if the rewarded corner was number 2 and the mouse makes drinking attempts in corner 3, it exhibits a DE. Drinking attempts in corners 2 or 4 do not contribute to it. The formula for DE is:

$$DE = \frac{\textit{Drinking attempts at clockwise position from previously correct corner}}{\textit{Total drinking attempts}}$$

As each challenge can comprise different types of errors, the CI is calculated to summarize the cognitive performance of each mouse through the challenge. Errors are taken into account according to their severity, with the most basic one (i.e., PE) being ′punished′ the most. The specific calculation for each challenge is available in Table 1.

As CE and DE are part of the overall PE, the place error rate had to be adjusted (PE_{adj}) for the calculation of the cognition index using the following formula:

$$PE_{adj} = PE - CE - DE$$

For the 4 types of errors, higher values indicate worse performance. However, for an index reflecting cognition, this seemed counter-intuitive and was therefore inverted. The overall CI formula is shown below:

$$CI = 1 - [(Weight_{PE} * PE_{adj}) + (Weight_{CE} * CE) + (Weight_{DE} * DE) + (Weight_{TE} * TE)]$$

For evaluating our challenges, learning curves were calculated for all. For each task and mouse, an ordered sequence of drinking attempts was extracted and classified as PE or not. The sequence of drinking attempts and cumulative successful drinking attempts were then used to create learning curves by linear regression. A dummy group, representing by-chance-performance, was created using a slope of 0.25.

Statistical analysis

The current version of IntelliR provides statistical analysis for all calculated variables for 2, 3 or 4 groups. The statistical pipeline was made using R 4.3.0[61] and base functions were used unless otherwise specified. Normality was tested through the Shapiro-Wilk test and equality of variance using Levene's test from the car R-package.[48]

When testing 2 groups, a variable with a parametric distribution is evaluated through a 2-sample t-test, with or without Welch's correction. Effect sizes are calculated with Cohen's d using the effectsize R-package.[49] For non-parametric variables, Wilcoxon Rank-Sum Test is used, and a note is made to indicate whether or not the variable had equal variance. Effect sizes are calculated with Cliff's delta using the effectsize R-package.[49]

When 3 or 4 groups are specified, normality and equality of variance are calculated as described above. For variables with a parametric distribution, One-Way ANOVA is used taking into account the result of Levene's test. Effect sizes are calculated using the Omega Squared test using the effectsize R-package.[49] Post-hoc comparisons are done using Tukey's range test (also known as Tukey's HSD), while for unequal variances, the Games Howell Test from the rstatix R-package was used, which employs Tukey's studentized range distribution for computing the p-values.[50] In both cases, effect sizes are calculated with Cohen's d using the effectsize R-package.[49]

For variables with non-parametric distributions and equal variances, the Kruskal-Wallis Rank-Sum Test is used. The epsilon-squared effect size is calculated according to Tomczak and Tomczak[62] with the following formula, where H is the Kruskal-Wallis test statistic and n is the sample size:

$$Epsilon - squared = \frac{H}{(n^2 - 1)/(n+1)}$$

Post-hoc statistics are calculated using Dunn's Test from the dunn.test R-package[51] and their effect sizes using Cliff's delta from the effectsize R-package.[49]

For non-parametric distributions with unequal variances, an Asymptotic K-Sample Fisher-Pitman Permutation Test is calculated using the coin R-package.[52] Unfortunately, no adequate effect size test was found for this case. Post-hoc comparisons are performed using simultaneous tests for general linear hypothesis with multiple comparisons of means using the glht function of the multcomp R-package.[53] Effect sizes are calculated using Hedges' g through the emmeans R-package.[54]

For the learning curves, global comparisons are calculated using analysis of covariance (ANCOVA), with specific group comparisons calculated through estimated marginal means contrasts using the emmeans R-package.[54]

Cell Reports Methods, Volume *5*

Supplemental information

A comprehensive and standardized pipeline for automated profiling of higher cognition in mice

Vinicius Daguano Gastaldi, Martin Hindermann, Justus B.H. Wilke, Anja Ronnenberg, Sahab Arinrad, Sabine Kraus, Anne-Fleur Wildenburg, Antonios Ntolkeras, Micah J. Provost, Liu Ye, Yasmina Curto, Jonathan-Alexis Cortés-Silva, Umer Javed Butt, Klaus-Armin Nave, Kamilla Woznica Miskowiak, and Hannelore Ehrenreich

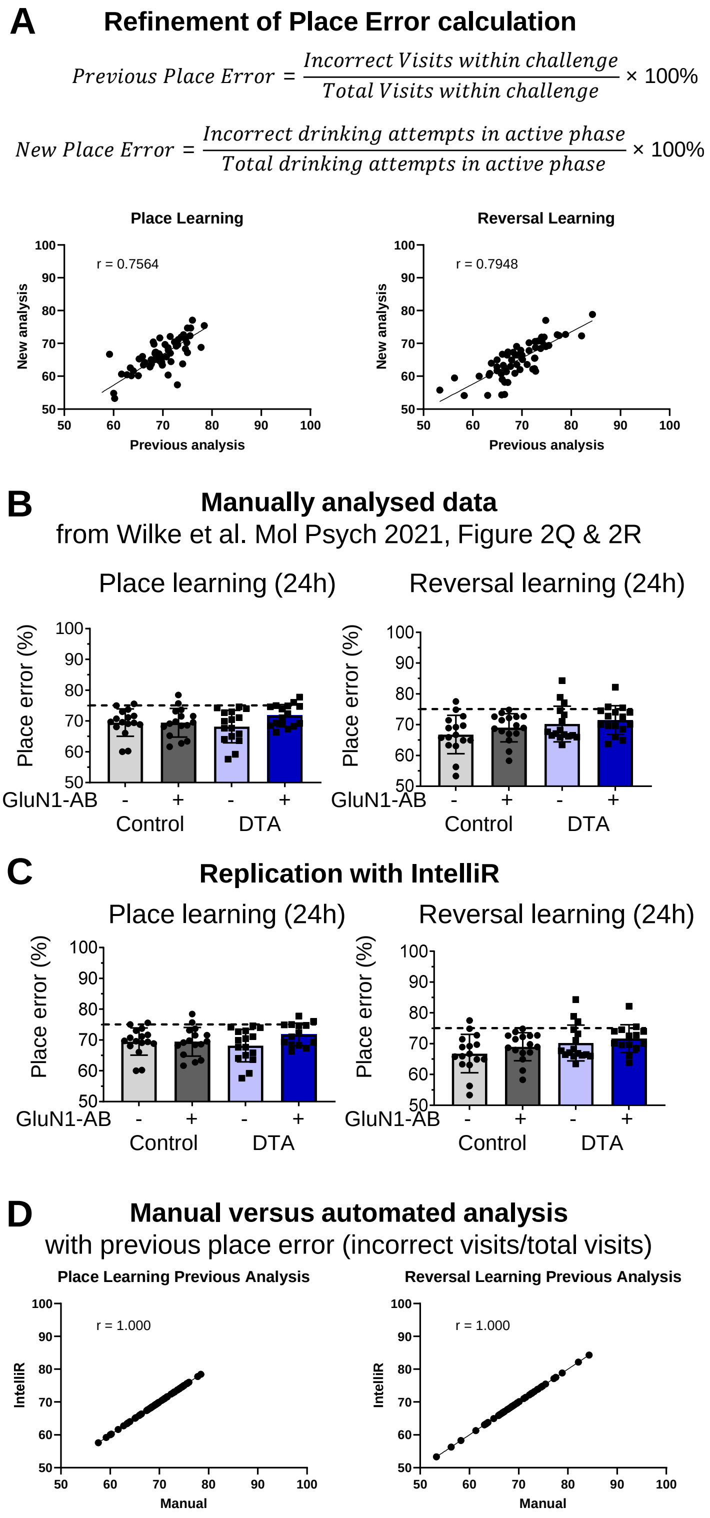

Supplementary Figure 1: Replication of manually analyzed IntelliCage data with IntelliR. (A) Comparison of the previous place error calculation used by Wilke et al. 2021 [S1], that was based on corner visits independent of nose pokes and the new place error calculated by IntelliR, that distinguished between exploratory visits and drinking attempts (visits with nose poke). While Pearson correlations of the previous and new place error calculation show substantial correlation between the old and new analysis (r=0.75-0.79), they also reveal differences, likely due to the exclusion of exploratory visits in the new Place Error. Hence, to test if automated data analysis yields the same results as manual analysis, we implemented the previous place error calculation into IntelliR and re-analyzed the data published by Wilke et al. 2021 [S1] **(B-C)**. **(D)** Pearson correlation analysis of manually and automatically analyzed IntelliCage data demonstrate perfect replication of manually analyzed data with IntelliR (r=1.0). Related to Figures 2, 4, and 5.

Reference:

[S1] Wilke, J.B.H., Hindermann, M., Berghoff, S.A., Zihsler, S., Arinrad, S., Ronnenberg, A., Barnkothe, N., Steixner-Kumar, A.A., Roglin, S., Stocker, W., et al. (2021). Autoantibodies against NMDA receptor 1 modify rather than cause encephalitis. Mol Psychiatry 26, 7746-7759. 10.1038/s41380-021-01238-3.
